# Supplementary figures and images for: A randomized trial to evaluate the impact of copra meal hydrolysate on gastrointestinal symptoms and gut microbiome
Source: PeerJ. 2021 Sep 15;9:e12158. doi: 10.7717/peerj.12158 (PMC8449532; doi:10.7717/peerj.12158)

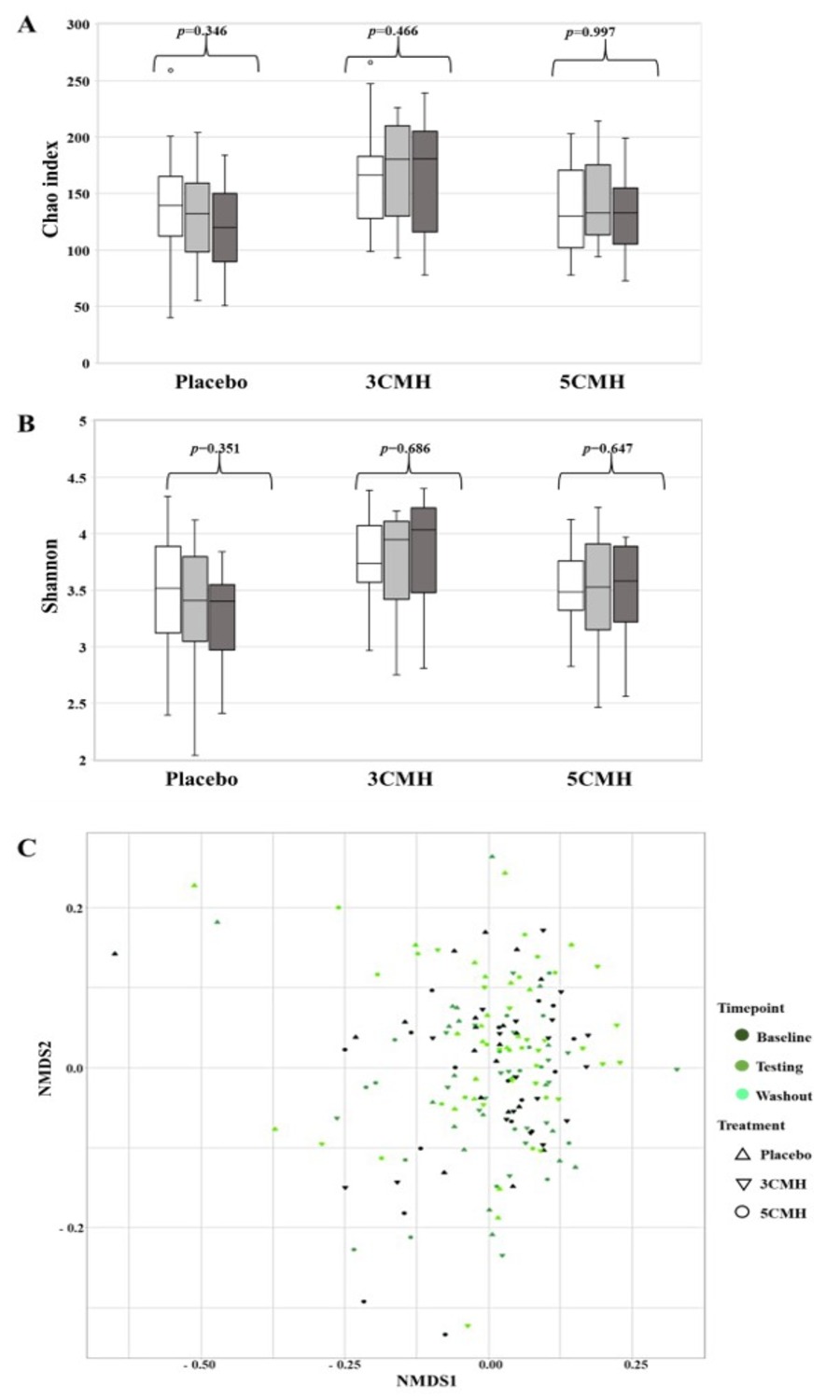

Supplement: Supplemental Information 1 — Alpha-diversity index: Chao-1 (A) and Shannon (B) from placebo, 3CMH 5CMH groups at baseline (white), testing period (gray) and washout period (black). Beta diversity of bacterial communities (C) in placebo, 3CMH and 5CMH at baseline, testing and washout period. Non-metric multidimensional scaling (NMDS) visualizations using the Bray Curtis metric separating. [file peerj-09-12158-s001.jpg]
