# Supplementary material for: A randomized trial to evaluate the impact of copra meal hydrolysate on gastrointestinal symptoms and gut microbiome
Source: PeerJ. 2021 Sep 15;9:e12158. doi: 10.7717/peerj.12158 (PMC8449532; doi:10.7717/peerj.12158)
Supplement: Supplemental Information 5 — Values are expressed as mean ± SD. *Significant difference between sample groups by the Kruskal–Wallis test (p ≤ 0.05). [file peerj-09-12158-s005.docx]

**Supplemental Table S4.**  **Defecating conditions and gastrointestinal symptoms at baseline, testing and washout period.**

|  | **Placebo** | **3CMH** | **5CMH** | ***p*-value** | | | |
| --- | --- | --- | --- | --- | --- | --- | --- |
|  |  |  |  | **P vs 3CMH vs 5CMH** | **P vs 3CMH** | **P vs 5CMH** | **3CMH vs 5CMH** |
| **Baseline** | | | | | | | |
| Defecation times per day | 1 ± 0.48 | 1.07 ± 0.51 | 0.98 ± 0.39 | 0.802 | 0.627 | 0.884 | 0.527 |
| Size of stool (determined as no. of ping-pong balls) | 2.5 ± 1.14 | 2.51 ± 1.25 | 2.43 ± 1.03 | 0.972 | 0.984 | 0.844 | 0.829 |
| Stool consistency (1: hard to 6: soft & watery) | 2.75 ± 0.93 | 3.2 ± 1.91 | 2.59 ± 1.04 | 0.345 | 0.302 | 0.705 | 0.161 |
| Stool color (1: yellow to 6: dark brown) | 2.82 ± 0.81 | 3.25 ± 1.31 | 2.8 ± 1.53 | 0.444 | 0.283 | 0.957 | 0.260 |
| Stool odor (1: weak to 6: very strong) | 2.68 ± 1.15 | 2.41 ± 1.24 | 2.29 ± 1.17 | 0.568 | 0.474 | 0.301 | 0.748 |
| Flatulate and bloating (0: none to 3: severe symptoms) | 0.81 ± 0.61 | 0.99 ± 0.56 | 0.79 ± 0.72 | 0.555 | 0.377 | 0.919 | 0.326 |
| Discomfort (0: good well-being to 3: discomfort) | 0.41 ± 0.4 | 0.45 ± 0.48 | 0.49 ± 0.59 | 0.880 | 0.823 | 0.615 | 0.780 |
| Flatulate/bloating and discomfort (0: none to 3: severe symptoms) | 0.61 ± 0.43 | 0.72 ± 0.49 | 0.64 ± 0.63 | 0.800 | 0.520 | 0.860 | 0.640 |
| **Testing** | | | | | | | |
| Defecation times per day | 1.11 ± 0.59 | 1.02 ± 0.45 | 0.99 ± 0.46 | 0.730 | 0.563 | 0.450 | 0.858 |
| Size of stool (determined as no. of ping-pong balls) | 3.25 ± 2.33 | 1.99 ± 0.84 | 2.55 ± 1.52 | 0.068 | 0.021* | 0.195 | 0.295 |
| Stool consistency (1: hard to 6: soft & watery) | 3.81 ± 1.96 | 3.46 ± 1.7 | 3.11 ± 1.85 | 0.487 | 0.547 | 0.233 | 0.550 |
| Stool color (1: yellow to 6: dark brown) | 3.69 ± 1.8 | 3.34 ± 1.39 | 3.15 ± 1.56 | 0.556 | 0.486 | 0.289 | 0.713 |
| Stool odor (1: weak to 6: very strong) | 3.43 ± 1.81 | 2.72 ± 1.54 | 2.81 ± 1.78 | 0.370 | 0.198 | 0.257 | 0.875 |
| Flatulate and bloating (0: none to 3: severe symptoms) | 0.9 ± 0.99 | 0.62 ± 0.55 | 0.83 ± 0.81 | 0.519 | 0.275 | 0.795 | 0.404 |
| Discomfort (0: good well-being to 3: discomfort) | 0.54 ± 0.8 | 0.45 ± 0.43 | 0.53 ± 0.72 | 0.889 | 0.653 | 0.942 | 0.705 |
| Flatulate/bloating and discomfort (0: none to 3: severe symptoms) | 0.72 ± 0.83 | 0.53 ± 0.46 | 0.68 ± 0.74 | 0.670 | 0.397 | 0.853 | 0.507 |
| **Washout** | | | | | | | |
| Defecation times per day | 1.01 ± 0.57 | 0.95 ± 0.4 | 0.84 ± 0.41 | 0.508 | 0.704 | 0.255 | 0.446 |
| Size of stool (determined as no. of ping-pong balls) | 3.12 ± 2.3 | 2.2 ± 1.42 | 2.28 ± 1.38 | 0.190 | 0.100 | 0.134 | 0.882 |
| Stool consistency (1: hard to 6: soft & watery) | 3.09 ± 1.73 | 3.11 ± 1.37 | 2.52 ± 1.49 | 0.391 | 0.964 | 0.246 | 0.228 |
| Stool color (1: yellow to 6: dark brown) | 3.46 ± 1.77 | 3.06 ± 1.24 | 2.71 ± 1.53 | 0.302 | 0.404 | 0.124 | 0.473 |
| Stool odor (1: weak to 6: very strong) | 3.26 ± 1.77 | 2.42 ± 1.17 | 2.36 ± 1.59 | 0.126 | 0.090 | 0.068 | 0.896 |
| Flatulate and bloating (0: none to 3: severe symptoms) | 0.72 ± 0.66 | 0.42 ± 0.41 | 0.59 ± 0.7 | 0.287 | 0.116 | 0.484 | 0.378 |
| Discomfort (0: good well-being to 3: discomfort) | 0.33 ± 0.39 | 0.23 ± 0.26 | 0.38 ± 0.61 | 0.587 | 0.512 | 0.718 | 0.311 |
| Flatulate/bloating and discomfort (0: none to 3: severe symptoms) | 0.52 ± 0.42 | 0.33 ± 0.32 | 0.48 ± 0.63 | 0.383 | 0.191 | 0.781 | 0.300 |

Values are expressed as mean ± SD

^*^Significant difference between sample groups by the Kruskal-Wallis test (*p* ≤ 0.05).
