# Supplementary material for: A randomized trial to evaluate the impact of copra meal hydrolysate on gastrointestinal symptoms and gut microbiome
Source: PeerJ. 2021 Sep 15;9:e12158. doi: 10.7717/peerj.12158 (PMC8449532; doi:10.7717/peerj.12158)
Supplement: Supplemental Information 9 — All values are expressed as median ± SD [file peerj-09-12158-s009.docx]

**Supplemental Table S8** Relative abundance of gut microbiome phyla for placebo, 3CMH and 5CMH groups at baseline, testing period and washout period.

|  | **Placebo** | | | | **3CMH** | | | | **5CMH** | | | |
| --- | --- | --- | --- | --- | --- | --- | --- | --- | --- | --- | --- | --- |
|  | **Baseline** | **Testing** | **washout** | ***p*-value** | **Baseline** | **Testing** | **washout** | ***p-value*** | **Baseline** | **Testing** | **washout** | **p-value** |
| Actinobacteria | 1.40 ± 5.62 | 2.37± 5.24 | 2.35± 6.12 | 0.241 | 0.95 ± 1.98 | 1.47± 1.87 | 2.25± 3.14 | 0.108 | 1.64 ± 7.13 | 2.22 ± 7.07 | 1.08± 3.05 | 0.585 |
| Bacteroidetes | 2.70 ± 7.14 | 4.45 ± 7.50 | 1.22 ± 4.61 | 0.803 | 3.67± 8.86 | 6.43 ± 10.49 | 4.03± 7.56 | 0.328 | 2.44± 7.15 | 7.78± 7.66 | 2.29± 4.14 | 0.435 |
| Firmicutes | 82.92 ± 14.21 | 78.63± 18.59 | 86.16± 13.73 | 0.446 | 85.89± 8.75 | 88.54 ± 14.18 | 84.93± 8.27 | 0.424 | 84.18± 10.20 | 83.34 ± 8.20 | 87.67 ± 10.94 | 0.683 |
| Lentisphaerae | 4.84 ± 6.70 | 1.60± 14.33 | 2.28 ± 6.29 | 0.833 | 1.99± 2.53 | 1.62± 5.85 | 1.71± 3.88 | 0.314 | 1.44 ± 2.52 | 1.54± 8.15 | 2.854± 10.14 | 0.242 |
| Proteobacteria | 4.84 ± 6.70 | 1.60 ± 14.33 | 2.28 ± 6.29 | 0.99 | 1.99± 2.53 | 1.62± 5.85 | 1.71± 3.88 | 0.351 | 1.44 ± 2.52 | 1.54± 8.15 | 2.85 ± 10.14 | 0.869 |
| Verrucomicrobia | 0.01 ± 13.43 | 0.06± 10.99 | 0 ± 6.99 | 0.375 | 0.11± 5.85 | 0.07± 4.75 | 0.08 ± 2.88 | 0.425 | 0 ± 2.49 | 0 ± 2.48 | 0 ± 0.71 | 0.564 |
| Firmicute/Bacteroidetes ratio | 33.99 ± 1578.08 | 15.07 ± 72.93 | 66.91± 303.63 | 0.263 | 23.95± 61.92 | 13.92± 20.89 | 21.70± 51.82 | 0.301 | 16.04 ± 93.73 | 13.34± 23.26 | 37.67 ± 359.11 | 0.760 |

All values are expressed as median ± SD
